# Development and Validation of a Novel Histone Acetylation-Related Gene Signature for Predicting the Prognosis of Ovarian Cancer

**DOI:** 10.3389/fcell.2022.793425

**Published:** 2022-02-18

**Authors:** Qinjin Dai, Ying Ye

**Affiliations:** ^1^ Guangzhou Women and Children’s Medical Center, Guangzhou Medical University, Guangzhou, China; ^2^ Department of Cardiothoracic Surgery, The Second Affiliated Hospital of Chongqing Medical University, Chongqing, China

**Keywords:** ovarian cancer, histone acetylation, gene signature, overall survival, predictive model

## Abstract

Histone acetylation is one of the most common epigenetic modifications, which plays an important role in tumorigenesis. However, the prognostic role of histone acetylation-regulators in ovarian cancer (OC) remains little known. We compared the expression levels of 40 histone acetylation-related genes between 379 OC samples and 88 normal ovarian tissues and identified 37 differently expressed genes (DEGs). We further explored the prognostic roles of these DEGs, and 8 genes were found to be correlated with overall survival (*p* < 0.1). In the training stage, an 8 gene‐based signature was conducted by the least absolute shrinkage and selector operator (LASSO) Cox regression. Patients in the training cohort were divided into two risk subgroups according to the risk score calculated by the 8-gene signature, and a notable difference of OS was found between the two subgroups (*p* < 0.001). The 8-gene risk model was then verified to have a well predictive role on OS in the external validation cohort. Combined with the clinical characteristics, the risk score was proved to be an independent risk factor for OS. In conclusion, the histone acetylation-based gene signature has a well predictive effect on the prognosis of OC and can potentially be applied for clinical treatments.

## Introduction

Ovarian cancer (OC) owns the highest mortality rate among all gynecological tumors (Kuroki and Guntupalli, 2020). Generally, most of the OC patients were at an advanced stage when being diagnosed, due to the lack of typical clinical symptoms and available screening biomarkers in the early stages (Krzystyniak et al., 2016). In the treatment phase, chemotherapy resistance is easy to develop, causing a high rate of recurrence (Christie and Bowtell, 2017). In spite of the therapeutic regimens that have been rapidly developed, the 5-years survival rate of OC has been poorly improved in decades. As the limited treatment methods, researchers have been searching for reliable markers to predict the prognosis of ovarian cancer and the therapeutic targets. Recently, human epididymis protein 4 (HE4) has been found to have a better predict role on recurrence in comparison to CA-125 (Scaletta et al., 2017). However, in clinical practice, either CA-125 or HE4 can only be used as a monitoring/predictive indicator while not as a therapeutic target; In addition, single-gene predictive models tend to have low specificities. Conversely, predictive models based on multiple genes often showed better predicting efficacies. With the increasing data of next-generation sequencing (NGS), a few systematic predictive models based on multiple genes have been established and revealed favorable accuracy. In recent years, a growing number of studies have found that multi-targeted combination therapies can help to improve the prognosis of tumor patients, thus it remains essential to identify new potential anti-tumor targets.

Epigenetic modifications are genetic modifications that cause heritable phenotypic changes in the expression of a gene without altering its DNA sequence, which has been proved to regulate the expression of tumor-related genes, and could be potential therapeutic targets (Cao and Yan, 2020). In eukaryotes, histones are bound to DNA to form chromatin, and the terminal amino acid residues can be covalently modified by methylation, acetylation, phosphorylation, ubiquitination, and adenosine diphosphate (ADP) glycosylation (Bennett and Licht, 2018). Among them, acetylation modification of the N-terminus of histone H3 and H4 lysine residues is the main histone modification associated with transcription, chromatin remodeling, and DNA expression and repair. Histone acetylation is a reversible dynamic equilibrium process, mediated by histone acetyltransferases (HAT) and histone deacetylases (HDAC) (Kaypee et al., 2016). In the presence of HAT, the acetyl is transferred to the N-terminal lysine residue of the histone, which counteracts the positive charge of the residue and allows the DNA conformation to unfold, resulting in the relaxation of the nucleosome structure and the activation of the transcription of the specific genes, whereas the function of HDAC is the opposite (Shen et al., 2015). HATs have been proved to be involved in the transformation of malignant tumors, not as direct tumor suppressors or oncogenes, but as acetyltransferases and transcriptional co-activators regulating the expression of key genes and proteins (Ell and Kang, 2013). HDACs include several family members and are currently one of the most studied anti-cancer targets. By inhibiting HDACs’ activity and promoting histone acetylation, histone deacetylase inhibitors (HDACis) can inhibit cancer cell growth, induce differentiation and apoptosis (Autin et al., 2019). Normally, HATs and HDACs dynamically regulate the processes in the histone acetylation, while the unbalance of acetylation level could contribute to cancer development. The HAT/HDAC inhibitors can affect gene expression by altering the acetylation level of histones in specific regions of chromatin, making them a new class of antitumor drugs with promising development and application, therefore, exploring the prognostic value of these histone deacetylase-related regulators is essential for the development of highly selective targeted drugs for a specific type of cancer.

However, most studies preferred to explore one single histone acetylation site or the specific role of HDACs (Zhou et al., 2018), ignoring the importance of HATs and sirtuin family proteins. For this reason, we performed a study to make a comprehensive understanding of the expression levels of the histone acetylation-related genes between OC and normal ovarian tissues and to explore the prognostic value of these regulators.

## Materials and Methods

### Data Sources

The transcriptome sequencing data of 379 OC patients and the corresponding clinical information were acquired from The Cancer Genome Atlas (TCGA) database (downloaded at https://portal.gdc.cancer.gov/). As lacking data of normal group, we combined the Genotype-Tissue Expression (GTEx) database to get the RNA sequencing data of ovarian tissue from 88 normal women (downloaded at https://xenabrowser.net). The GTEx Research Consortium study collected more than 7,000 autopsy samples from 449 humans, and the 88 normal ovarian tissues were all obtained from previously healthy human donors. A validation cohort with the RNA-seq data and the clinical features of 380 OC patients were obtained from the Gene Expression Omnibus (GEO) database (https://www.ncbi.nlm.nih.gov/geo/, GSE140082). The read count values in each database were all downloaded as fragments per kilobase million (FPKM), and to minimize the batch effects, we applied the “Combat” function in the “SVA” R package. Moreover, we employed the “Scale” function to further normalize the expression level of each gene before cross-validations.

The 40 histone acetylation-related regulators were retrieved from a review (Cheng et al., 2019), and they were shown in Supplementary Table S1.

### Identification of Differently Expressed Genes Between Tumor and Normal Tissues

In this phase, the TCGA and the GTEx datasets were merged, and before comparisons, the expression data were both normalized by FPKM. The DEGs between tumor and normal tissues were identified by applying the “limma” R package (with *FDR* < 0.05). The heatmaps of DEGs were accomplished by using the “heatmap” R package and the spearman correlation analysis was conducted by the “reshape2” R package.

### Development and Validation of the Histone Acetylation-Related Gene Signature

To assess the prognostic value of each histone acetylation-related DEG, we combined the gene expression data and the corresponding overall survival time and survival status information of each patient in the TCGA cohort (training cohort). The univariate Cox regression model was utilized to screen the prognosis-related genes. Those genes with *p* < 0.1 were chosen for developing the prognostic signature by applying the Least absolute shrinkage and selection operator (LASSO) cox regression model provided from the “glmnet” R package. Finally, 8 genes with nonzero coefficients were decided by the minimum criteria and the risk score of the gene signature was calculated by the formula: $Risk\;Score=\overset{n}{\underset{i}{\sum }}X_{i}\times Y_{i}$ (X: coefficient of each gene, Y: gene expression level). Referred to the median risk score of the training cohort, patients were divided into low- and high-risk groups. The Kaplan-Meier analysis was utilized to compare the OS time and survival possibility between the low- and high-risk populations. The principal components analysis (PCA) and the t-distributed stochastic neighbor embedding (t-SNE) were performed based on the 8-gene signature by applying the “ggpolt2” and the “Rtsne” R packages. To assess the sensitivity and the specificity of the risk score, we constructed a 3-years ROC curve by applying the “survival”, “survminer” and “timeROC” R packages. In the validation phase, patients from the GEO cohort were also classified into low- and high-risk populations according to the median risk score from the training cohort, and the two populations were compared to validate the gene signature.

### Independent Prognostic Analysis

We employed the univariate and multivariable cox regression models (applying the “survival” R package) to assess the independent prognostic value of the risk score. Moreover, clinical features of age and tumor stage were carried out into the regression models to identify whether the risk score was independently correlated with OS. The Nomogram conducted by the “survival” and the “rms” packages were presented to show the results of the predicting model. Besides, the calibration curve was plotted to evaluate the consistency between ideal and actual predicting outcomes.

### Functional Enrichment Analysis

**FIGURE 1:**
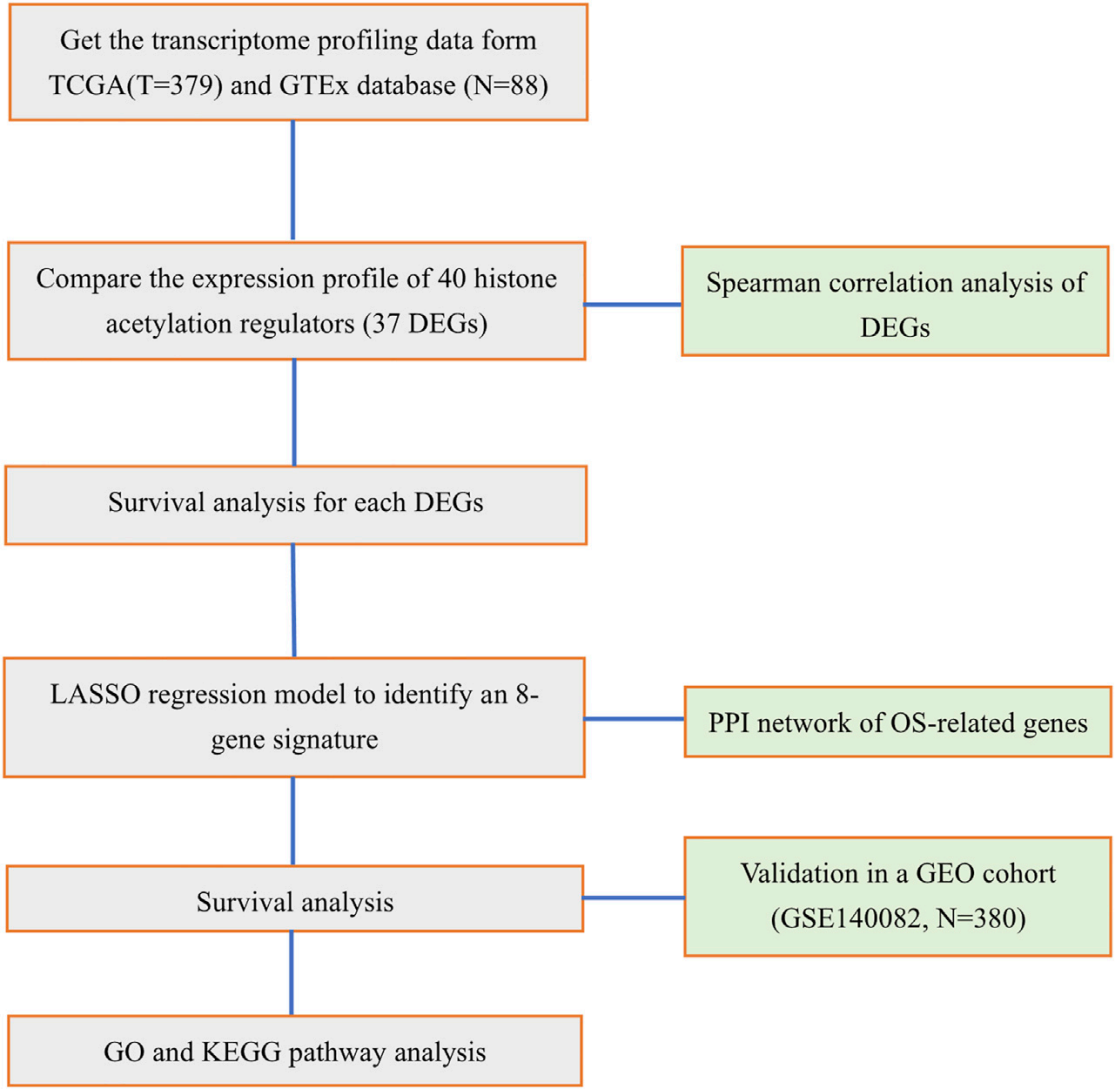
Workflow diagram for the study.

Based on the risk score, patients in the TCGA cohort were stratified into low- and high-risk groups. According to the criteria of |log_2_ FC|>1, FDR<0.05, the DEGs between the two risk groups were screened out. The Gene Ontology (GO) and the Kyoto Encyclopedia of Genes and Genomes (KEGG) analyses were performed by the “cluster Profiler” R package. Moreover, to make comparisons of the infiltrating scores of immune cells and the activities of immune pathways between low- and high-risk groups, we employed the single-sample gene set enrichment analysis (ssGSEA), which was accomplished by the “gsva” R package. The full workflow diagram is shown in Figure 1.

### Statistical Analysis

When comparing the gene expression level between two groups, the Student’s t-test was utilized, while the Pearson chi‐square test was applied to compare the categorical variables. The Kaplan-Meier analysis was used for comparing the OS time and survival possibilities between low- and high-risk subgroups. To assess the independent prognostic value of the risk model, the univariate and multivariable cox regression models were applied. All statistical analyses were completed by the R software (version 4.0.2).

## Results

### Identification of Differently Expressed Genes Between Normal and Tumor Tissues

**FIGURE 2:**
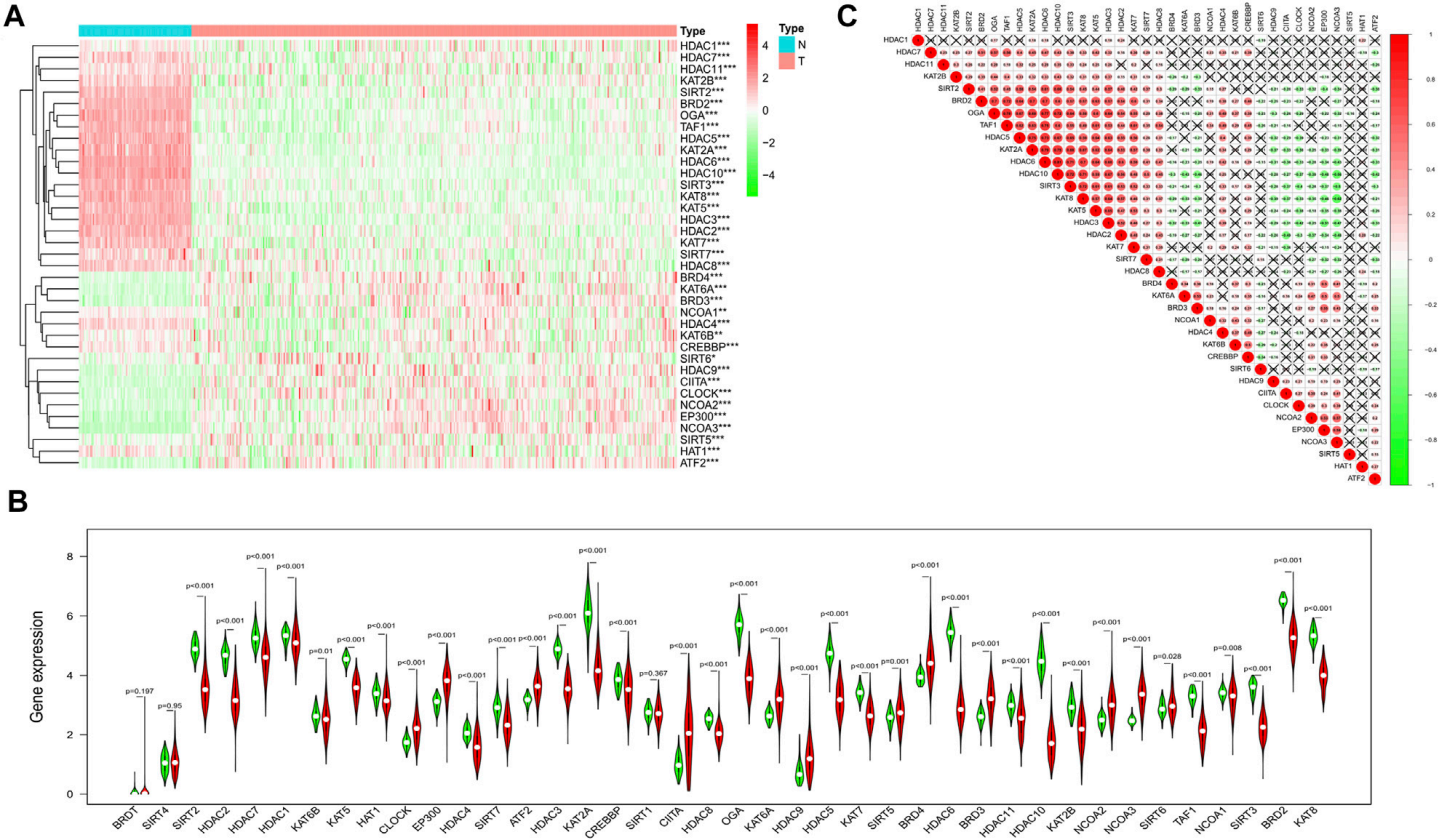
Identification of the DEGs between the normal and ovarian cancer tissues. **(A)** Heatmap of all the DEGs in normal and tumor tissues (green: low-expression; Red: high expression). **(B)** The violin plot for all the histone acetylation-related regulators (Red: tumor tissues; Green: normal ovarian tissues). **(C)** Spearman correlation analysis for the 37 DEGs (Red: positive correlation, Green: negative correlation, the absolute values of correlation coefficient less than 0.10 were marked with "×").

We combined the TCGA and the GTEx datasets and got the gene expression data of 379 tumors and 88 normal ovarian tissues. The gene expression levels of the 40-histone acetylation regulators were all compared and finally 37 DEGs were identified. Among them, 25genes were at a lower expression level in the tumor group, while the other 12 genes were enriched. The heatmap for each DEG and the violin plot for all 40 genes were presented in Figures 2A,B. To better understand the correlations of these DEGs, a spearman correlation analysis was performed (Figure 2C).

### Development of a Prognostic Gene Signature in the Training Set

**FIGURE 3:**
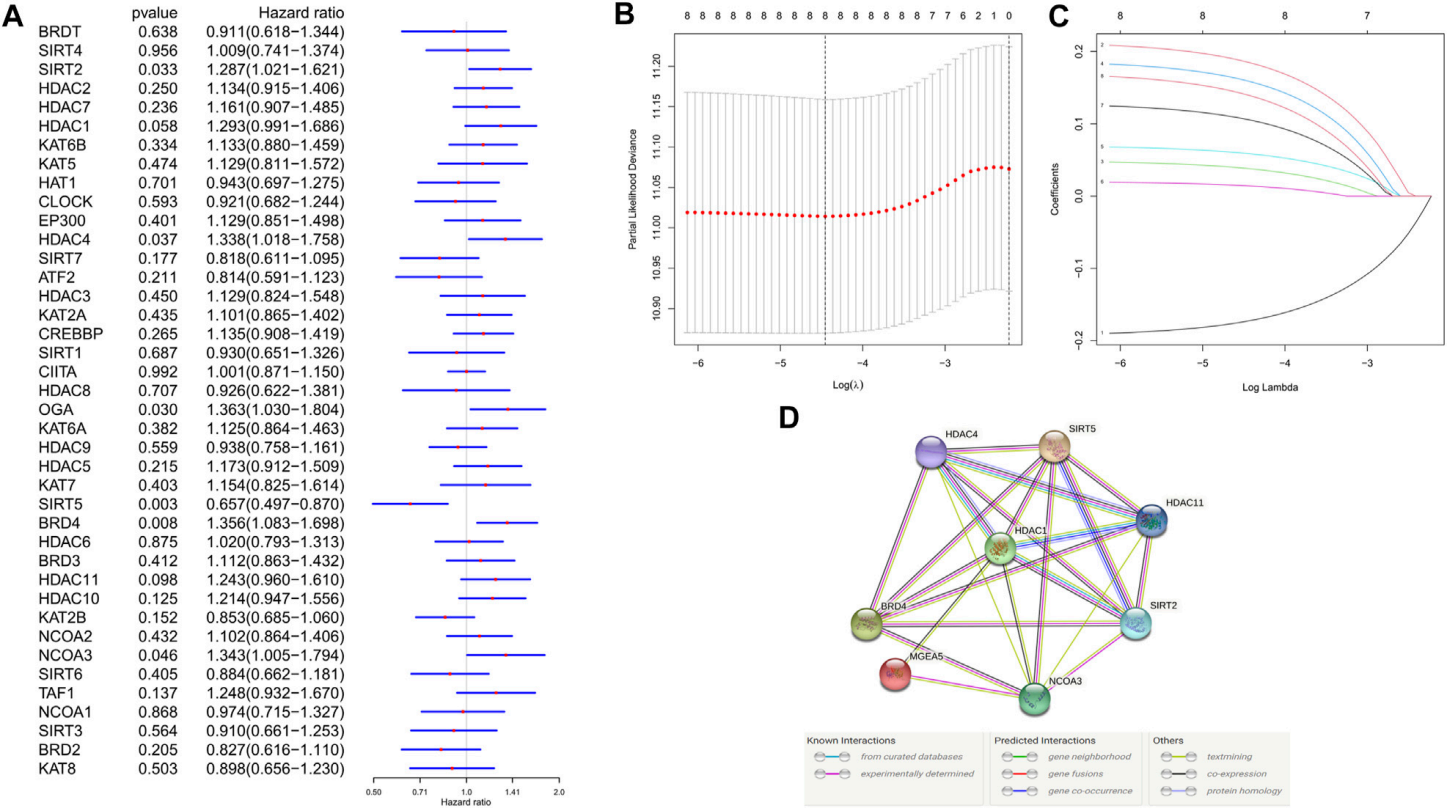
Development of risk signature in the training cohort. **(A)** Univariate cox regression analysis of OS for each histone acetylation-related regulator, and 8 genes were identified with *p* < 0.1. **(B)** Cross-validation for tuning the parameter selection in the LASSO regression. **(C)** LASSO regression and the coefficients of the 8 OS-related genes. **(D)** PPI network showing the interactions between the OS-related genes.

**FIGURE 4:**
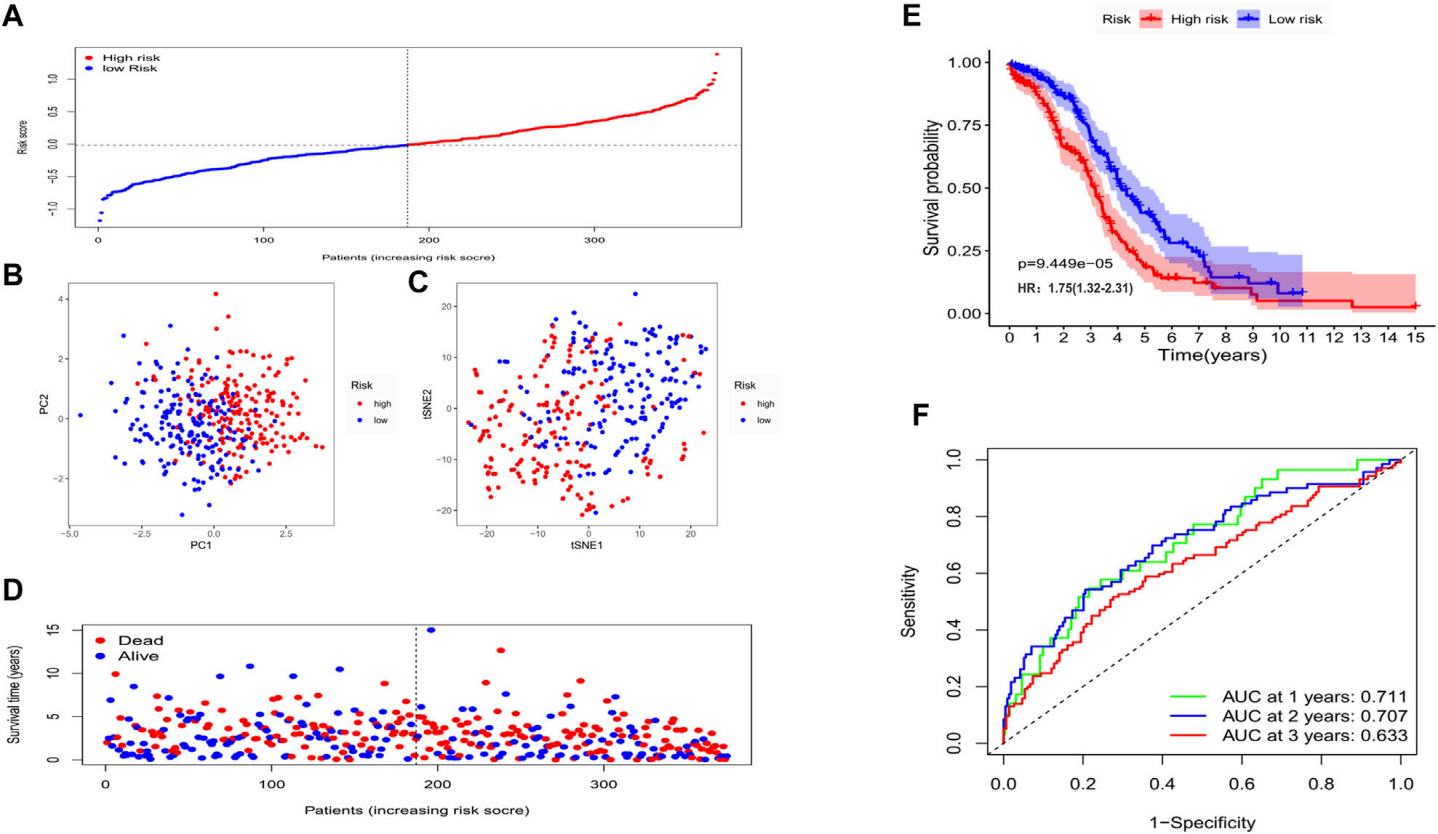
The prognostic value of the gene signature in the training set. **(A)** The distribution of patients based on the median risk score. **(B)** PCA plot for OCs based on the risk score. **(C)** The t-SNE analysis based on the risk score. **(D)** The survival status for each individual (left of the dotted line: low-risk population; right of the dotted line: high-risk population). **(E)** Kaplan-Meier curves for the OS of patients between the high- and low-risk groups. **(F)** Time-dependent ROC curves demonstrated the predictive efficiency.

In the training cohort (TCGA cohort), we analyzed the prognostic value of each histone acetylation-related DEG by applying the univariate cox regression model. Totally, 8 survival-related genes were picked out (with the *p* value < 0.1) for further analysis, they were *SIRT5*, *BRD4*, *OGA*, *SIRT2*, *HDAC4*, *NCOA3*, *HDAC1*, and *HDAC11* (Figure 3A). We employed the least absolute shrinkage and selection operator (LASSO) Cox regression model to construct a risk signature and ultimately, 8 genes were all retained according to the optimum λ value (Figures 3B,C). According to each gene’s coefficient, the risk score was calculated as follow: risk score = -0.173* *SIRT5(exp)* + 0.185*BRD4(exp) + 0.038*OGA(exp) + 0.159*SIRT2(exp) + 0.059*HDAC4(exp) + 0.014*NCOA3(exp) + 0.106*HDAC1(exp) + 0.140*HDAC11(exp). To explore the interactions of the 8 genes, a protein-protein interactions (PPI) network was conducted by the Search Tool for the Retrieval of Interacting Genes (STRING), version 11.0 (https://string-db.org/) (Figure 3D). According to the median risk score, 187 patients were treated as the low-risk group, while the other 187 were regarded as the high-risk group (5 patients without corresponding survival information were excluded) (Figure 4A). The PCA and the t-SNE analysis revealed that patients with different risks were tended to separate in two directions (Figures 4B,C). We can observe from the distribution graph that high-risk patients were at lower survival rates and shorter survival times than those with low-risk (Figure 4D). Similarly, the Kaplan-Meier curve also demonstrated a notable shorter OS time and lower survival possibility in the high-risk population (HR: 1.75, 95% CI: 1.32–2.31, *p* < 0.001, Figure 4E). The predictive accuracy of the risk score was assessed by the time-dependent receiver operating characteristic (ROC) analysis, and we found the area under the curve (AUC) was 0.711 for 1 year, 0.707 for 2 years, and 0.633 for 3 years (Figure 4F).

### Validation of the Gene Signature in a Gene Expression Omnibus Cohort

**FIGURE 5:**
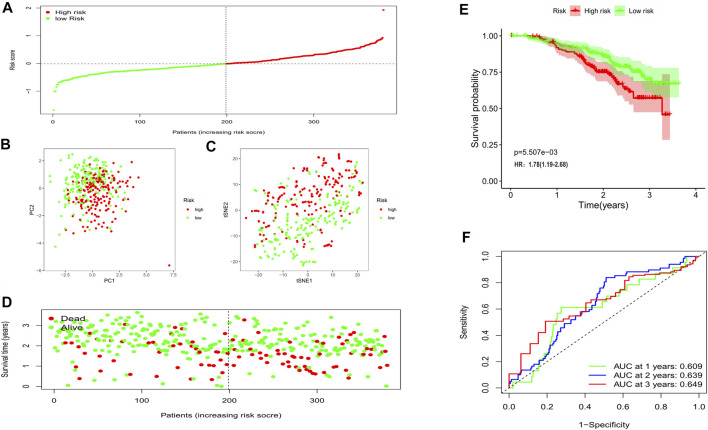
Validation of the risk model in the external cohort. **(A)** Distribution of patients in the validation cohort based on the median risk score of the training cohort. **(B)** The PCA plot for OCs. **(C)** The t-SNE analysis based on the risk score. **(D)** The survival status for each patient (left of the dotted line: low-risk population; right of the dotted line: high-risk population). **(E)** Kaplan-Meier curves for the OS. **(F)** Time-dependent ROC curves for OCs.

In this phase, 380 OC patients from a GEO cohort were regarded as the validation set. By applying the median risk score of the training cohort, 199 patients were treated as the low-risk group, while the other 181 were at high risk (Figure 5A). Consistently, the different risk populations could also be separated into two clusters when applying the PCA and the t-SNE analysis (Figures 5B,C). Notable higher mortality and a shorter OS time were discovered in the high-risk patients (Figure 5D); Besides, the survival analysis also indicated a lower survival possibility in the high-risk group (HR: 1.78, 95% CI: 1.19–2.68, *p* < 0.01, Figure 5E). The ROC curve showed the risk score could be a favorable predictor in the external dataset as well, and the AUC was 0.609 for 1 year, 0.639 for 2 years, and 0.649 for 3 years.

### Independent Prognostic Value of the Risk Score

**FIGURE 6:**
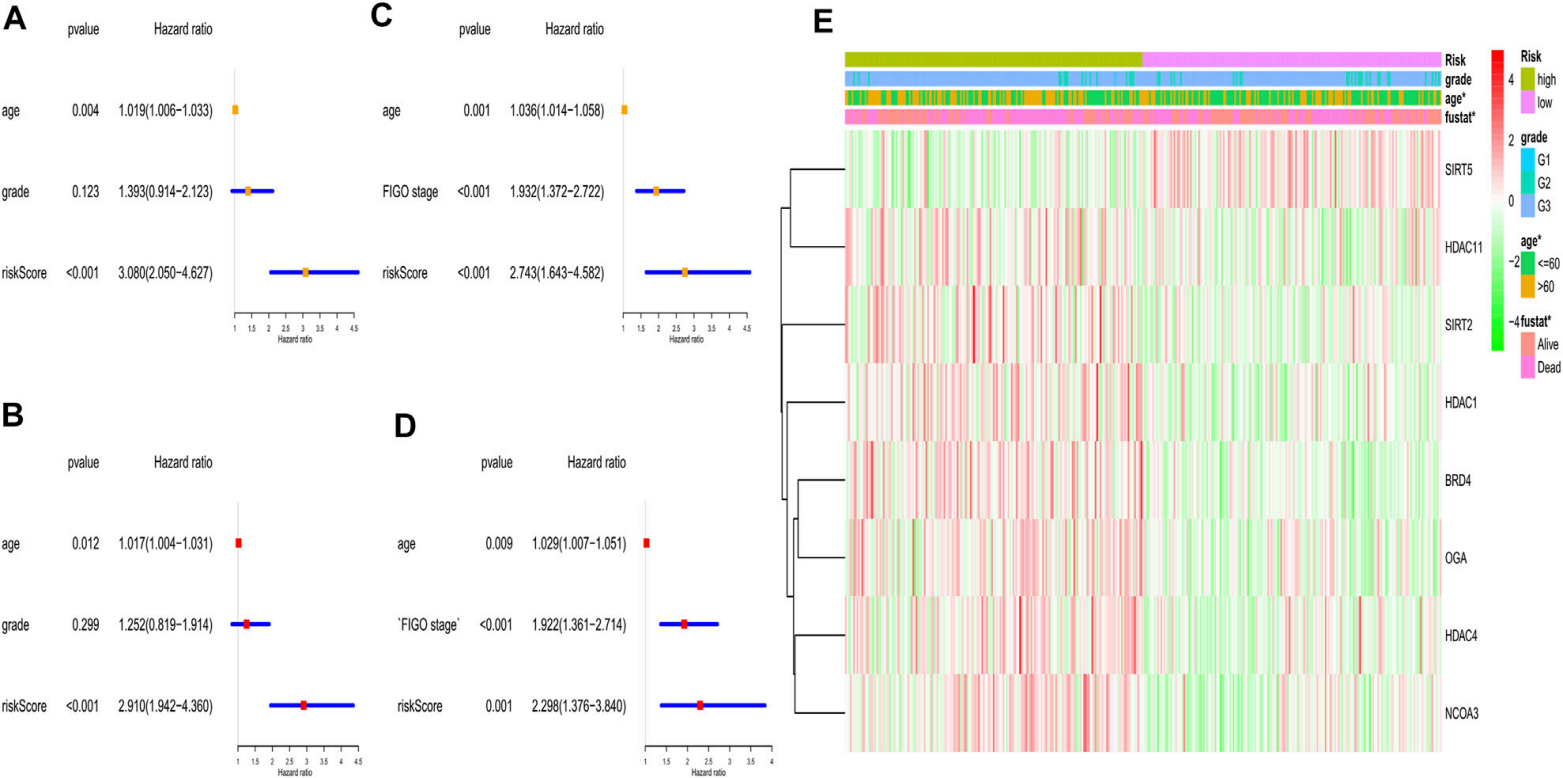
Univariate and multivariate Cox regression analyses for the risk score. **(A)** Univariate analysis for the TCGA (training) cohort. **(B)** Multivariate analysis for the TCGA (training) cohort. **(C)** Univariate analysis for the GEO (validation) cohort. **(D)** Multivariate analysis for the GEO (validation) cohort. **(E)** Heatmap for the gene expression combined with clinical features in the TCGA (training) cohort.

**FIGURE 7:**
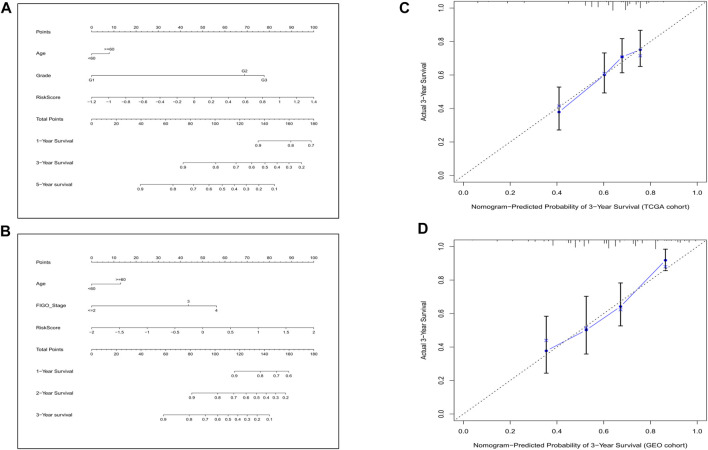
Construction of the predictive model. **(A)** The prognostic model to predict the OS in the training cohort. **(B)** The prognostic model to predict the OS in the validation cohort. **(C)** Calibration curves for the OS nomogram model in the training set. **(D)** Calibration curves for the OS nomogram model in the validation set.

To explore whether the gene signature-based risk score could be an independent prognostic factor, we applied the univariate and multivariable Cox regression models. In the training cohort, univariate analysis showed the age and the risk score were correlated with the prognosis (Figure 6A), furthermore, in the multivariable model, it indicated that the 2 factors could be independent predictors for prognosis (HR for risk score: 2.910, 95% CI: 1.942–4.360, *p* < 0.001, Figure 6B). In the validation cohort, the risk score was also found to be an independent risk factor (HR: 2.298, 95% CI: 1.376–3,840, *p* = 0.001, Figures 6C,D). Moreover, a heatmap combined with the clinical features in the training cohort implied that the age and the survival status were significantly different between low- and high-risk subgroups (Figure 6E). These independently associated risk factors were used to build a risk estimation nomogram in both the training and the validation cohort (Figures 7A,B). The calibration curve for the survival status at 3 years showed that the nomogram performed well in the two cohorts (Figures 7C,D).

### Functional Analyses of Differently Expressed Genes Between the Two Risk Groups

**FIGURE 8:**
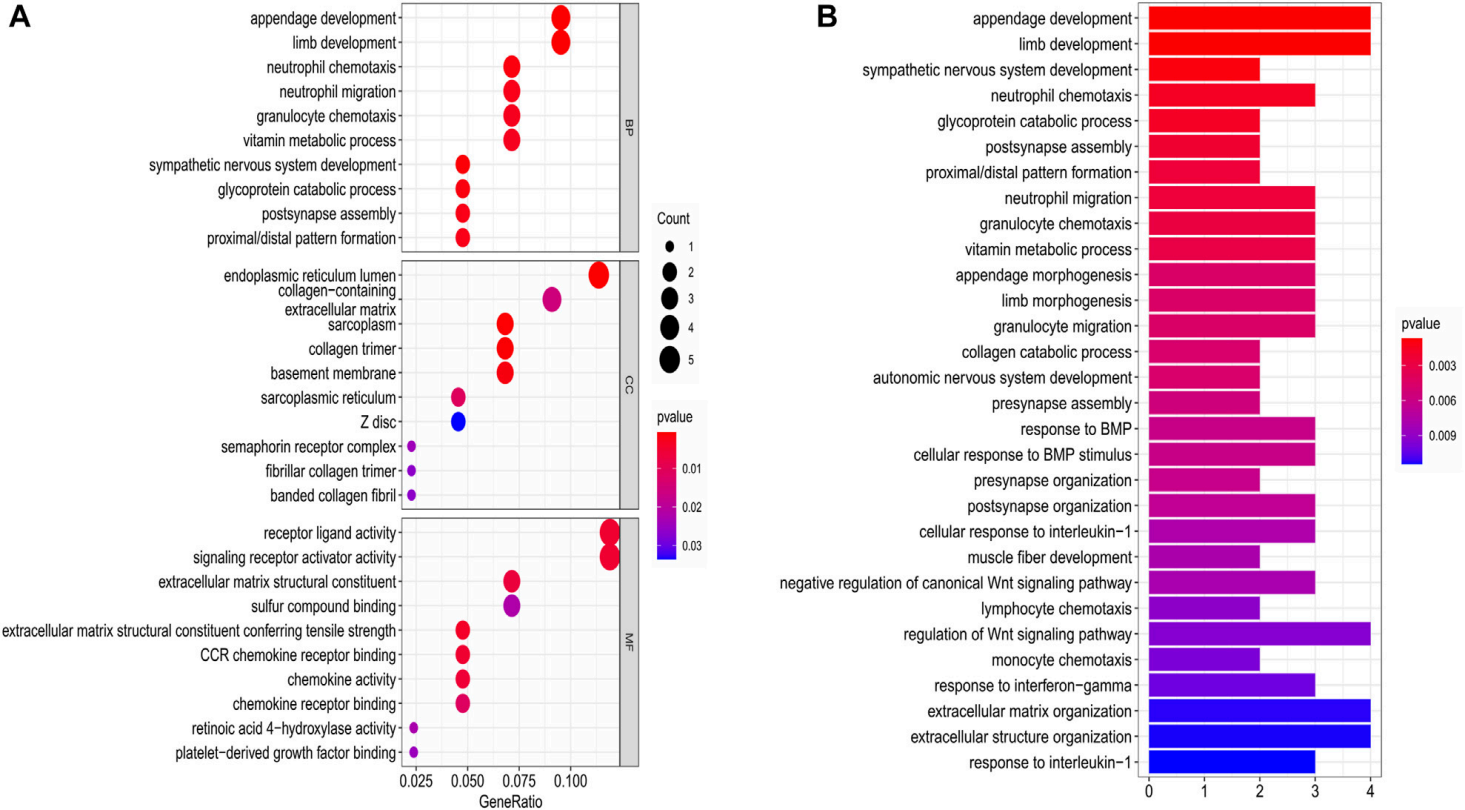
Functional analysis based on the DEGs between the two-risk groups in the training cohort. **(A)** Bubble graph for GO enrichment. **(B)** Bar plot for KEGG pathways (the length of the bar presents the amount of gene enrichment).

The DEGs between the low- and high-risk subgroups were identified by the screening criteria with *p*-value < 0.05 and |log_2_FC| >1. In total, 55 DEGs were screened out, and 18 of them were negatively correlated with the risk scores (Supplementary Table S2). To explore the potential functions of these DEGs, the GO (bubble plot) and KEGG pathway (bar plot) analyses were performed (Figures 8A,B). The results indicated that these DEGs were correlated with the development of organs, chemotaxis of immune cells, and regulation of signaling pathways. Notably, it’s worth exploring the associations between OC and Wnt signaling pathway, as some of the DEGs (*RSPO4*, *NOTUM*, and *SFRP5*) were involved in this canonical signaling pathway.

### Comparison of Immune Activity Between the Two Risk Groups

**FIGURE 9:**
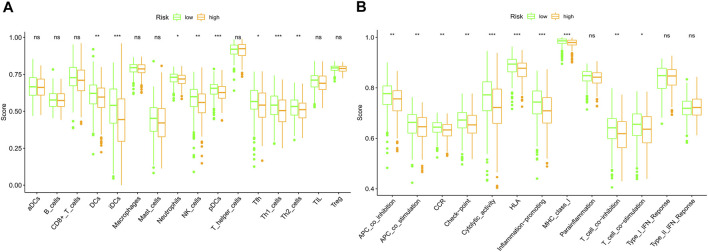
Comparison of the immune cells and immune pathways between low (green)- and high (orange)-risk subgroups based on the ssGSEA. **(A)** Compare the enrichment scores of immune cells. **(B)** Compare of the immune-related pathways. (ns: not significant; *p < 0.05; **p < 0.01; ***p < 0.001).

As the functional analyses revealed that lots of DEGs were related to the chemotaxis of immune cells, we next compared the infiltrating scores of 16 types of immune cells and the activity of 13 immune pathways. The results indicated that the dendritic cells (DCs), induced dendritic cells (iDCs), neutrophils, natural killer cells (NK cells), plasmacytoid dendritic cells (pDCs), follicular helper T cells (Tfh), and T-help 1/2 cells were at lower levels of infiltrations in the high-risk subgroup (Figure 9A). Meanwhile, we discovered that most of the immune pathways were with decreased activity in the high-risk population (Figure 9B).

## Discussion

In this study, we systematically investigated the gene expression levels of the currently known 40 histone acetylation-related regulators in ovarian cancer and found most of them expressed differently between tumor and normal tissues, indicating that these regulators play important roles in the genesis and development of OC. We applied the LASSO Cox regression model to construct an 8-gene signature, which was validated to be an independent risk factor for the OS of OCs. The functional analyses manifested that the DEGs between the two groups which were divided by the signature were enriched in the organ development, immune cell chemotaxis, and regulation of signaling pathways.

Histone acetylation modifications can affect the cell cycle, cell differentiation, and apoptosis. An imbalance in acetylation will lead to abnormal gene expression and alterations in important physiological functions, leading to tumorigenesis (Farria et al., 2015). According to the specific functions, these histone acetylation regulators could be divided into 3 categories: the “writers”, act as histone acetyltransferases; the “readers”, help to recognize acetyl-lysine sites; and the “erasers”, in charge of histone deacetylation (Cheng et al., 2019). However, their interactions and the correlations with OC patients’ OS remain largely unknown. In the 8-gene based risk signature, *OGA* and *NCOA3* belong to the “writers”, *BRD4* functioned as the “readers” and the “erasers” contain *SIRT2*, *SIRT5*, *HDAC1*, *HDAC4*, *HDAC11*. Except for *SIRT5*, other 7 genes were identified as the risk factor in predicting the OS of OC patients (with HR > 1). *OGA* owns the ability to acetylate histones, as the carboxyl terminus contains the structural domain of histone acetyltransferase. *OGA* was reported to have a higher expression level in poorly differentiated laryngeal tumor cells and was associated with a poor prognosis (Starska et al., 2015). While *OGA* was found to be negatively correlated with tumor progression in breast cancer (Krześlak et al., 2012). In ovarian cancer, inhibition of *OGA* would lead to *p53* stabilization and increase its nuclear localization and indicated *OGA* is a potential therapeutic target (de Queiroz et al., 2016). *NCOA3* (*KAT13B*) is a member of the Src/p160 nuclear receptor co-activation family, owning the histone acetyltransferase activity and promoting gene transcription. *NCOA3* was initially found to be highly expressed in breast cancer, and it was later discovered to be amplified in many other malignant diseases (Gojis et al., 2010). *NCOA3* was also found to be a marker for platinum resistance in ovarian cancer (Palmieri et al., 2013). *BRD4* is a histone acetylation site reader, binds in hyper-acetylated chromatin regions, acts as a nucleation center for the assembly of large protein complexes, promotes RNA-PolII activity, and stimulates transcription initiation and elongation (Devaiah et al., 2016). The inhibitor of *BRD4* has great promise for cancer therapy, it competes with acetylated residues to bind to the *BRD4* bromodomain, thereby reducing RNA-PolII flux and blocking transcription of key oncogenes (Donato et al., 2017). In dealing with ovarian cancer, *BRD4* inhibitors could re-sensitize drug-resistant cancer cells to anti-cancer drugs (Andrikopoulou et al., 2021). *SIRT2* can catalyze the deacetylation of histone H3 and H4, leading to dense chromatin curl and consequent inhibition of gene transcription and expression (Budayeva and Cristea, 2016). *SIRT2* up-regulation may contribute to cisplatin sensitivity in OC cell lines and may be positively correlated with longer OS time (Wang et al., 2020; Zeng et al., 2020). However, our study implied that *SIRT2* is a risk factor for OC patients, and the conclusion is consistent with Teng’s study (Teng and Zheng, 2017). Thus, the specific mechanisms of *SIRT2* in ovarian cancer still need further exploration. *SIRT5* has a weaker ability to deacetylate histones compared to other members of the sirtuins family (He et al., 2012). *SIRT5* was the unique gene identified in this study that was positively associated with a better prognosis. As the multiple protein modification functions of *SIRT5*, the protective role on ovarian cancer remains for further investigations. *HDAC1* is mainly involved in the deacetylation of histones. The correlations between *HDAC1* and the prognosis of malignant diseases are not clear since the controversial role of *HDAC1* in diverse types of cancer. *HDAC1* over-expression was discovered to prolong the OS time in Asian breast cancer patients (Qiao et al., 2018), while it was a risk factor for patients with lung cancer (Cao et al., 2017). Knocking down of *HDAC1* has been reported to enhance the sensitivity to cisplatin-based chemotherapy in ovarian cancer (Liu et al., 2018), indicating that *HDAC1* is a potential therapeutic target. *HDAC4* can deacetylate lysine residues at sites K9, K14, K18, and K23 of histone H3 and sites K5, K8, K12, and K16 of H4 (Wang et al., 2014). *HDAC4* is aberrantly expressed in a variety of cancer cells and tissues and may play a role in cancer development. Besides, *HDAC4* was also found to be involved in the process of platinum resistance in ovarian cancer (Stronach et al., 2011). *HDAC11* is responsible for the deacetylation of core histones and is a key factor in the regulation of transcription and the cell cycle (Licciardi et al., 2013; Liu et al., 2020). It has been reported that the deletion of *HDAC11* leads to cell death in colon, prostate, breast, and ovarian cancer cell lines (Deubzer et al., 2013). However, a recent study indicated that down-regulation of *HDAC11* would promote tumor metastasis from lymph nodes in breast cancer (Leslie et al., 2019). As the double-edged roles of *HDAC11*, and the little evidence in ovarian cancer treatment, furthermore in-depth studies about *HDAC11* should be taken into consideration. Notably, half of the poor prognosis-related genes own the ability of histone deacetylation (*SIRT2*, *HDAC1*, *HDAC4*, *HDAC11*). As a prior study reported, HDACs and the sirtuins family proteins are often over-expressed in malignancies, and promote tumor progression (Hai et al., 2021). However, the specific mechanisms of HDACs or sirtuins were quite different among tumors, thus to identify survival-related HDACs would help to facilitate targeted therapies towards ovarian cancer. Moreover, HDACs and HATs dynamically regulated the processes in the histone acetylation, thereby co-influencing transcription of oncogenes and tumor suppressor genes, while lots of studies only focused on the predictive roles of HDACs, neglecting the importance of HATs. Our work made a systematic study on the prognostic roles of these histone acetylation regulators, providing insights and novel molecular targets of epigenetic therapy towards ovarian cancer.

When the GO and KEGG pathway analyses were performed, we found that a variety of receptor-ligand interactions, as well as the immune cells-chemotaxis biological processes and pathways, were enriched. Moreover, lots of DEGs were correlated with the regulation of the Wnt signaling pathway, and this signal pathway was proved to contribute to the cisplatin resistance and weaken the anti-tumor immunity in OC (Doo et al., 2020; He et al., 2021). In this study, the top 3 Wnt signaling pathway-related genes were *RSPO4*, *NOTUM*, and *SFRP5*. R-spondin 4 (*RSPO4*) is an agonist of the Wnt signaling pathway, and was elevated about 2.5 folds in the high-risk population in our study, indicating that *RSPO4* may promote ovarian cancer progression through activation of the Wnt pathway. *NOTUM* (notum palmitoleoyl-protein carboxylesterase) acts as a negative regulator of the Wnt pathway, however, it was highly enriched in the high-risk group (about 6-folds up-regulated). A recent study demonstrated that *NOTUM* may be a novel efficacious target in treating colorectal cancer (Flanagan et al., 2021), so it’s worth initiating explorations of *NOTUM* in ovarian cancer as lacking data. *SFRP5* (secreted frizzled related protein 5) can negatively regulate the Wnt pathway, and is regarded as a tumor suppressor. Our study found that *SFRP5* was unexpectedly up-regulated (3-folds) in the high-risk group, and the reason may be caused by the epigenetic silencing of *SFRP5* in the high-risk group (Su et al., 2010). In addition, as the Wnt pathway plays a pivotal role in organ development (Liu et al., 2022), it is not difficult to explain why lots of the DEGs are associated with organ development in our study.

According to the analyses of immune cells and pathways, we can speculate that these histone acetylation-related genes may affect the components of the tumor immune microenvironment and could regulate the immune-related signaling pathways. These findings may provide new targets for anti-tumor immunotherapies.

## Conclusion

In conclusion, our study implied that histone acetylation is closely correlated with ovarian cancer and we developed a novel prognostic model of 8 histone acetylation-related genes. This model was proved to be independently associated with OS in OC patients, supplying a new strategy for predicting the prognosis and treating with OC.

## Data Availability

The datasets presented in this study can be found in online repositories. The names of the repository/repositories and accession number(s) can be found in the article/Supplementary Material.

## Abbreviations

OC, ovarian cancer; LASSO, least absolute shrinkage and selection operator; TCGA, The Cancer Genome Atlas database; GEO, gene expression omnibus; GTEx, genotype-tissue expression; GO, gene ontology; KEGG, kyoto encyclopedia of genes and genomes; DEGs, differentially expressed genes; PCA, principal component analysis; t-SNE, t-distributed stochastic neighbor embedding.
