# Supplementary material for: Development and Validation of a Novel Histone Acetylation-Related Gene Signature for Predicting the Prognosis of Ovarian Cancer
Source: Front Cell Dev Biol. 2022 Feb 18;10:793425. doi: 10.3389/fcell.2022.793425 (PMC8894724; doi:10.3389/fcell.2022.793425)
Supplement: Supplementary file 1 [file Table1.DOCX]

| Genes | Full Names |
| --- | --- |
| ATF2 | activating transcription factor 2 |
| BRD2 | bromodomain containing 2 |
| BRD3 | bromodomain containing 3 |
| BRD4 | bromodomain containing 4 |
| BRDT | bromodomain testis associated |
| CIITA | class II major histocompatibility complex transactivator |
| CLOCK | clock circadian regulator |
| CREBBP | CREB binding protein |
| EP300 | E1A binding protein p300 |
| HAT1 | histone acetyltransferase 1 |
| HDAC1 | histone deacetylase 1 |
| HDAC2 | histone deacetylase 2 |
| HDAC3 | histone deacetylase 3 |
| HDAC4 | histone deacetylase 4 |
| HDAC5 | histone deacetylase 5 |
| HDAC6 | histone deacetylase 6 |
| HDAC7 | histone deacetylase 7 |
| HDAC8 | histone deacetylase 8 |
| HDAC9 | histone deacetylase 9 |
| HDAC10 | histone deacetylase 10 |
| HDAC11 | histone deacetylase 11 |
| KAT2A | lysine acetyltransferase 2A |
| KAT2B | lysine acetyltransferase 2B |
| KAT5 | lysine acetyltransferase 5 |
| KAT6A | lysine acetyltransferase 6A |
| KAT6B | lysine acetyltransferase 6B |
| KAT7 | lysine acetyltransferase 7 |
| KAT8 | lysine acetyltransferase 8 |
| NCOA1 | nuclear receptor coactivator 1 |
| NCOA2 | nuclear receptor coactivator 2 |
| NCOA3 | nuclear receptor coactivator 3 |
| OGA | O-GlcNAcase |
| SIRT1 | sirtuin 1 |
| SIRT2 | sirtuin 2 |
| SIRT3 | sirtuin 3 |
| SIRT4 | sirtuin 4 |
| SIRT5 | sirtuin 5 |
| SIRT6 | sirtuin 6 |
| SIRT7 | sirtuin 7 |
| TAF1 | TATA-box binding protein associated factor 1 |
