# Supplementary material for: Development and Validation of a Novel Histone Acetylation-Related Gene Signature for Predicting the Prognosis of Ovarian Cancer
Source: Front Cell Dev Biol. 2022 Feb 18;10:793425. doi: 10.3389/fcell.2022.793425 (PMC8894724; doi:10.3389/fcell.2022.793425)
Supplement: Supplementary file 2 [file Table2.DOCX]

| gene | lowMean | highMean | logFC | pValue | fdr |
| --- | --- | --- | --- | --- | --- |
| RSPO4 | 2.625517 | 6.18523 | 1.236225 | 3.23E-07 | 5.50E-06 |
| FLNC | 2.299619 | 5.063328 | 1.138691 | 9.68E-06 | 8.50E-05 |
| MIR4449 | 1.824826 | 0.844129 | -1.11222 | 1.13E-05 | 9.60E-05 |
| RNY1 | 1.698649 | 0.789631 | -1.10514 | 0.000264 | 0.001288 |
| LINC02575 | 0.698227 | 1.916269 | 1.456532 | 1.84E-06 | 2.20E-05 |
| MMP12 | 4.074049 | 1.728203 | -1.23719 | 0.001464 | 0.005241 |
| MMP1 | 5.674928 | 2.579516 | -1.1375 | 0.000473 | 0.002071 |
| SST | 367.7246 | 131.3544 | -1.48516 | 0.000518 | 0.002225 |
| AVPR2 | 0.808697 | 1.797126 | 1.152021 | 0.000154 | 0.000838 |
| LRRC4B | 0.787572 | 1.762386 | 1.162047 | 9.14E-07 | 1.27E-05 |
| PLXNA4 | 0.618996 | 1.737305 | 1.488849 | 1.06E-06 | 1.43E-05 |
| IGLON5 | 1.854043 | 5.110739 | 1.462857 | 6.15E-07 | 9.31E-06 |
| NKAIN4 | 1.342292 | 3.28398 | 1.290747 | 0.000183 | 0.000965 |
| FGF19 | 0.680876 | 1.728072 | 1.343699 | 0.000997 | 0.003835 |
| RNA5SP202 | 9.942993 | 3.221415 | -1.62599 | 0.000512 | 0.002205 |
| INA | 1.775262 | 4.029368 | 1.182521 | 1.71E-07 | 3.29E-06 |
| NCCRP1 | 1.404561 | 4.606545 | 1.713566 | 1.59E-08 | 4.99E-07 |
| VNN1 | 2.190791 | 0.992071 | -1.14294 | 0.001637 | 0.005749 |
| SLC25A3P1 | 4.996255 | 2.014505 | -1.31042 | 7.10E-10 | 4.29E-08 |
| ARHGAP40 | 0.637718 | 1.425325 | 1.160299 | 5.55E-07 | 8.55E-06 |
| COL9A3 | 1.769211 | 3.982671 | 1.17063 | 1.35E-08 | 4.37E-07 |
| MIR7152 | 0.726479 | 1.975736 | 1.443398 | 1.48E-06 | 1.86E-05 |
| NOTUM | 2.812893 | 18.11182 | 2.686805 | 0.005141 | 0.014518 |
| CCL20 | 16.55894 | 5.994132 | -1.46599 | 1.01E-06 | 1.37E-05 |
| RNVU1-18 | 13.39728 | 4.164907 | -1.68558 | 0.009261 | 0.02349 |
| COL2A1 | 0.914305 | 3.717337 | 2.023523 | 0.000825 | 0.003288 |
| MUC4 | 0.575162 | 1.627893 | 1.500966 | 3.35E-06 | 3.56E-05 |
| LRP4 | 1.135981 | 2.915693 | 1.3599 | 1.06E-05 | 9.12E-05 |
| TMEM100 | 9.133077 | 4.246169 | -1.10494 | 0.007146 | 0.018947 |
| RNU1-83P | 2.082872 | 0.953281 | -1.1276 | 0.001114 | 0.004187 |
| RNA5S9 | 1.434919 | 0.577571 | -1.3129 | 0.000337 | 0.001554 |
| LINP1 | 3.515521 | 1.282406 | -1.45489 | 0.000125 | 0.000709 |
| NLRP7 | 1.010151 | 2.961547 | 1.55178 | 0.003186 | 0.009816 |
| SFRP5 | 1.013619 | 2.951696 | 1.542029 | 0.000226 | 0.001144 |
| HOXA9 | 0.57913 | 1.49086 | 1.364186 | 0.000978 | 0.003772 |
| UPK2 | 2.56718 | 6.470186 | 1.333623 | 0.000477 | 0.002082 |
| CD22 | 2.555578 | 5.720417 | 1.162471 | 6.81E-07 | 1.01E-05 |
| RBPJL | 0.16361 | 2.341696 | 3.839223 | 0.001503 | 0.005363 |
| CPLX2 | 1.413288 | 3.031585 | 1.101017 | 0.020718 | 0.044917 |
| HIST1H1C | 164.2379 | 69.26013 | -1.24569 | 3.36E-08 | 9.05E-07 |
| KRTDAP | 0.832985 | 2.02366 | 1.280605 | 0.000458 | 0.002015 |
| ZIC2 | 1.434184 | 5.392691 | 1.910776 | 0.004896 | 0.013953 |
| TKTL1 | 1.949402 | 7.655013 | 1.973373 | 0.008642 | 0.022158 |
| COL26A1 | 3.57623 | 7.835457 | 1.131578 | 0.00305 | 0.009466 |
| AC003035.2 | 1.506508 | 0.642545 | -1.22934 | 1.18E-09 | 6.36E-08 |
| ANKLE1 | 1.02813 | 2.3109 | 1.168433 | 6.05E-10 | 3.82E-08 |
| TAS1R3 | 0.56602 | 1.464868 | 1.371845 | 3.34E-09 | 1.42E-07 |
| GFRA3 | 0.6636 | 3.224814 | 2.28083 | 0.000248 | 0.001225 |
| FARSA-AS1 | 0.470397 | 2.732987 | 2.538528 | 0.008283 | 0.021368 |
| KCNG1 | 1.625 | 3.502287 | 1.107858 | 1.51E-07 | 3.00E-06 |
| UBD | 5.750875 | 2.56695 | -1.16373 | 5.05E-06 | 5.00E-05 |
| CYP2W1 | 0.588564 | 1.752862 | 1.574441 | 0.002875 | 0.00902 |
| RYR1 | 1.178056 | 2.774699 | 1.235923 | 2.17E-12 | 6.05E-10 |
| CCL13 | 2.044174 | 0.704487 | -1.53687 | 3.25E-05 | 0.000232 |
| THBS4 | 1.644697 | 3.64239 | 1.147063 | 0.007851 | 0.020466 |
